# A novel role for mitochondrial fission in macrophages: trained innate immunity induced by beta-glucan

**DOI:** 10.1038/s41423-023-01017-w

**Published:** 2023-04-17

**Authors:** Anna Brichkina, Hans-Uwe Simon

**Affiliations:** 1 Institute of Systems Immunology, Center for Tumor Biology and Immunology, Philipps University of Marburg, 35043 Marburg, Germany; 2 Member of the German Center for Lung Research (DZL), Philipps University of Marburg, 35043 Marburg, Germany; 3 Laboratory of Molecular Immunology, Institute of Fundamental Medicine and Biology, Kazan Federal University, 420012 Kazan, Russia; 4 Institute of Pharmacology, University of Bern, 3010 Bern, Switzerland; 5 Institute of Biochemistry, Brandenburg Medical School, 16816 Neuruppin, Germany

**Keywords:** Lipid signalling, Monocytes and macrophages

In a recent paper published in Nature Immunology, Ding et al. provided new insights into the mechanism of how trained innate immunity could eradicate cancer. The authors demonstrated that yeast-derived whole beta-glucan particles (WGP) increased the responsiveness of lung interstitial macrophages to tumor-derived factors associated with the subsequent inhibition of tumor metastasis through enhanced cytotoxicity to cancer cells. The authors identified the metabolic sphingolipid–mitochondrial fission axis in WGP-trained macrophages as the key pathway responsible for this phenomenon and categorized it as a mechanism of trained innate immunity [1].

Traditionally, the innate and adaptive immune systems are differentiated by their specificity and memory capacity. For a long time, it has been assumed that immunological memory is an exclusive hallmark of the adaptive immune response. On the other hand, innate immune cells have not been seen as cells that can retain a memory phenotype. In recent years, however, this paradigm has shifted: emerging evidence suggests that certain microbial stimuli and endogenous ligands induce durable changes in the function of innate immune cells that increase their responsiveness upon secondary stimulation. This process has been termed “trained innate immunity” or “trained immunity” [2]. After the first contact with a trained immunity-inducing stimulus, susceptible cells undergo metabolic, epigenetic and/or transcriptomic reprogramming, which results in increased responsiveness to a secondary insult [3, 4].

Trained innate immunity has been primarily described in monocytes and macrophages [3] and later in granulocytes [5]. These innate immune cells share the ability to recognize and respond to a broad repertoire of stimuli; however, most research on trained innate immunity has focused on the Bacille Calmette-Guérin (BCG) vaccine, a weakened version of *Mycobacterium bovis*, and fungal beta*-*glucans from *Candida albicans*, *Trametes versicolor* or *Saccharomyces cerevisiae*. Trained innate immunity has been explored in the treatment of infectious and inflammatory diseases, whereas enabling trained immunity as a therapeutic strategy for cancer has only recently emerged. For instance, BCG vaccination has been shown to have antitumor effects on bladder cancer, melanoma, lymphoma, and leukemia. Although beta-glucan has also been reported to induce an antitumor effect on primary subcutaneous tumors [5–7], the exact mechanism by which the antitumor response is elicited by trained innate immune cells to control cancer progression is not well understood. Therefore, the role of trained immunity in cancer and its therapeutic potential remain unclear.

**Fig. 1:**
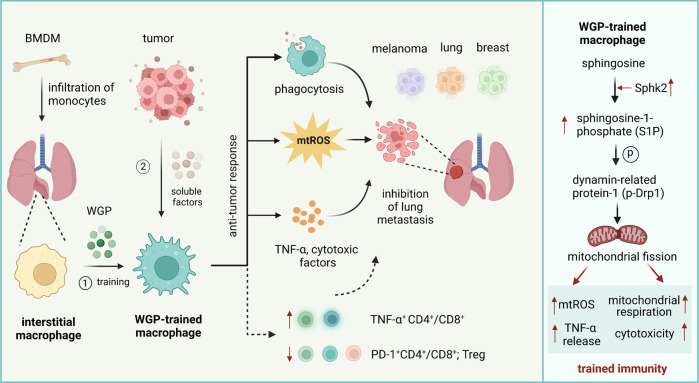
Macrophages trained with whole beta-glucan particles (WGP) inhibit tumor metastasis. Interstitial lung macrophages originating from the bone marrow can be trained with WGP in vivo and develop increased responsiveness to tumor cells or soluble tumor-derived factors. As a result, trained macrophages inhibit lung metastasis due to enhanced TNF-α production, increased phagocytosis and cytotoxicity to tumor cells and the modulation of T-cell responses. Innate immune training is mediated by enhanced synthesis of sphingolipids and the subsequent accumulation of S1P in macrophages, resulting in Drp1-regulated mitochondrial fission, which is required for increased mitochondrial ROS production and respiration, as well as increased cytotoxicity and TNF-α production

The group of Jun Yan from the University Louisville School of Medicine performed a comprehensive study to investigate trained immunity induced by yeast-derived WGP in macrophages and its potential therapeutic relevance for metastatic disease [1]. The authors used an experimental setup to study lung cancer and showed that WGP-treated mouse macrophages and human monocytes exhibited a trained enhanced response upon direct contact with tumor cells or exposure to tumor-derived soluble factors, such as macrophage migration inhibitory factor (MIF). Importantly, WGP could also induce trained immunity in vivo, increasing the production of myeloid cells in the bone marrow without systemic inflammation in tissues. For instance, WGP-treated mice or mice with reconstituted bone marrow from WGP-trained mice had prolonged survival and significantly reduced tumor burdens in the lungs after intravenous injection of lung cancer cells or in K-ras^LA1^ mice with spontaneous development of lung cancer. Interestingly, WGP-mediated training and metastasis control were explicitly associated with the functions of lung interstitial macrophages originating from the bone marrow but not alveolar macrophages and did not depend on neutrophils or B and T cells, which has been elegantly demonstrated by using neutralizing and depletion approaches in several tumor models. However, WGP-mediated training enhanced T-cell responses in tumor-bearing mice due to a reduced proportion of regulatory T cells, decreased frequencies of PD-1-positive CD4^+^ and CD8^+^ T cells and high production of T-cell-derived TNF-α. In addition, adoptive transfer of in vitro WGP-trained bone marrow-derived macrophages (BMDMs) into naive mice that were subsequently transplanted with lung cancer cells significantly reduced the tumor burden in lungs, suggesting a potential use of trained BMDMs as adoptive cell therapy in cancer. To further test potential clinical implications, the authors used multiple metastatic disease mouse models and demonstrated that melanoma and breast cancer metastasis in lungs and the dissemination of lymphoma cells in liver were substantially reduced in WGP-treated mice, emphasizing the systemic benefit of trained immunity to effectively control lung tumorigenesis and metastasis (Fig. 1).

Ding et al. performed a very detailed search for the molecular mechanism underlying WGP-mediated training in macrophages. They observed that WGP increased the production of TNF-α in lung interstitial macrophages and enhanced their phagocytic capacity and cytotoxicity to tumor cells. Interestingly, previously published pathways involved in inducing trained immunity, including the mTOR/HIF-1α or Nlrp3 inflammasome and IL-1β/IL-1R pathways [4], did not play a role in WGP-mediated training of macrophages. Instead, the sphingolipid-mediated mitochondrial fission pathway was responsible for WGP-induced trained immunity in macrophages and the subsequent control of metastasis. The metabolite sphingosine-1-phosphate (S1P) enhanced respiratory capacity and mtROS production in WGP-trained macrophages and increased their differentiation, migration and survival. Interestingly, dynamin-related protein-1 (Drp1) was required for these functional responses and activated by S1P. The presence of the S1P – Drp1 pathway leading to mitochondrial fission has previously been described in T cells [8]. These findings suggest that mitochondrial fission is essential for WGP-induced trained immunity of macrophages, resulting in the prevention, at least partially, of metastatic disease (Fig. 1).

In summary, Ding et al. discovered a novel mechanism for trained immunity in macrophages induced by WGP with implications for the treatment of primary and metastatic tumors. This strategy might be suitable for patients who require adjuvant therapies to prevent the occurrence of lung metastasis. Currently, however, it remains unclear whether WGP-trained immunity can also be effective against the dissemination of cancer cells in other organs. Given the numerous and diverse sources and processing of beta-glucan, one of the greatest expected challenges is the standardization of the molecules suitable for clinical use. On the other hand, increased mitochondrial fission and respiratory capacity can be achieved by specific drugs [9]. Moreover, the accumulation of S1P might also be achieved by the use of doxorubicin [10]. Such pharmacological approaches might be interesting future strategies for the induction of innate memory in macrophages, which could be manufactured off-the-shelf for adoptive cell therapy against cancer.
